# The multistep road to ventilator-associated lung abscess: A retrospective study of *S*.*aureus* ventilator-associated pneumonia

**DOI:** 10.1371/journal.pone.0189249

**Published:** 2017-12-20

**Authors:** Roman Mounier, David Lobo, Julia Voulgaropoulos, Mathieu Martin, Bouziane Aït-Mamar, Valérie Bitot, Paul-Henri Jost, Ron Birnbaum, Biba Nebbad, Fabrice Cook, Gilles Dhonneur

**Affiliations:** 1 Department of Anesthesia and Surgical Intensive Care, Henri Mondor University Hospital of Paris, Paris XII School of Medicine, Créteil, France; 2 Department of Anesthesia and Surgical Intensive Care, Bichat-Claude-Bernard University Hospital of Paris, Paris 12 School of Medicine, Paris, France; 3 Department of Microbiology, Henri Mondor University Hospital of Paris, Paris XII School of Medicine, Créteil, France; University of Rochester Medical Center, UNITED STATES

## Abstract

**Object:**

We observed some cases of lung abscess (LA) in ICU patients suffering *S*.*aureus* ventilator-associated pneumonia (*S*.*aureus*-VAP). We aimed to assess which of the host and/or bacteria-related features are associated with LA.

**Methods:**

We conducted a retrospective study from January 2009 to July 2013 in a trauma surgical ICU within a teaching hospital. All adult patients presenting with *S*.*aureus*-VAP were included. We compared two groups of patients according to the formation or not of LA concomitantly to *S*.*aureus*-VAP.

**Results:**

Seventy-nine *S*.*aureus*-VAP patients, predominantly males (85%) of rather young age (mean [SD]: 35yr [21–64]) with severe trauma (initial Simplified Acute Score II = 42 [32–52]) related-ICU admission, were included. Among them, 10 (14%) developed LA. Patient’s characteristics significantly associated with LA development were: a younger age (p = 0.003), road traffic accidents admission (p = 0.017), head injury (p = 0.002), lower Glasgow Coma Scale (p = 0.009), blunt chest trauma (p = 0.01) pneumothorax (p = 0.01) and lung contusions (p = 0.002). No microbiological factors were significantly associated with LA formation. Abscesses were mostly bilateral, ≥5 cm of diameter and with a posterior location.

**Conclusions:**

Our results do not favor a specific virulence of *S*.*aureus*, but rather highlight the role of multiple insults to the lung, promoting LA formation. Despite a similar severity score, patients with LA had more serious trauma, combining severe both chest and head insults.

## Introduction

*Staphylococcus aureus* is one of the most common pathogen in human disease [1,2]. It is the first pathogen responsible of endocarditis and osteo-articular infection, and the second bacterial specie identified in bacteriemia. Most patients with *S*.*aureus* related infection are elders and have serious underlying comorbidities [2,3]) *S*.*aureus* may carry various virulence factors leading to resistance to phagocytose by human immune cells, such as biofilm formation or the ability to penetrate into cells. Frequently, *S*.*aureus* participates to polymicrobial infection.

*S*.*aureus* is responsible for about 2% of community-acquired pneumonia but around 20% of ventilation acquired pneumonia (VAP), with a mortality rate ranging from 30% to 80% [2,4,5]. In some instances, *S*.*aureus* has been shown to disrupt the alveolo-capillar barrier and destroy the lung parenchyma [6]. A particular *S*.*aureus* related community-acquired pneumonia, which affects young and immunocompetent patients, was described. The clinical feature is spectacularly intense and associated high early mortality rate. Necropsy usually identifies diffuse, necrotizing, hemorrhagic processes combined to lung parenchymal abscesses. In these circumstances of necrotizing community-acquired pneumonia (NCAP), microbiological investigation may reveal a particular virulence of *S*.*aureus* related to carriage and expression of the Panton-Valentine Leukocidin (PVL) gene [4,7].

Interestingly, we observed in our trauma ICU unit a particular pattern of necrotizing VAP secondary to *S*.*aureus* infection (*S*.*aureus*-VAP), also occurring in young patients and characterized by LA formation. However, the pattern of such VAP differed from that of the *S*.*aureus*-NCAP. First, it developed under mechanical ventilation. Second, the development of lung injury was rather torpid. Finally, the outcome seemed to be more favorable.

The aim of the present study was to assess risk factors for LA development during *S*.*aureus*-VAP, and to describe the characteristics of its particular entity.

## Methods

### Patients

The study was conducted in a trauma and surgical intensive care unit from a single teaching university hospital. We retrospectively included all consecutive adult patients who experienced VAP due to *S*.*aureus*, from January 2009 to July 2013.

We excluded from this analysis (i): pneumonias that were not associated to mechanical ventilation; (ii) patients with preexisting (ICU admission) major lung insult (e.g.; important emphysema or history of LA), (iii) patients suffering from α1-antitrypsin deficiency or cystic fibrosis, (v) VAP without microbiological documentation, (vi) all situations when futility of care within the first 48h following diagnosis was anticipated, leading to withholding active treatment and providing palliative care only, (vii) and death within the first 48h (timeframe being insufficient for abscess formation, death was therefore due to another cause or to a too strong immunity response).

The Institutional Ethical Committee approved the retrospective study plan (Comité Ethique et Aide à la Décision Médicale, n°2014–05, Hôpital Claude Galien). The relatives of all patients admitted in the trauma ICU were informed that unaddressed data collected from recorded files may be used for clinical research. They were also advised that without a written notification that they refused the use of their relative’s information, their agreement would be implicit. In case of a positive ICU outcome, we attempted to reach oral consents given by the patient was conducted.

### Definitions

To establish the diagnosis of **pneumonia**, at least two of the following supportive clinical signs were required in the presence of new or progressive pulmonary infiltrates in radiographic assessments: a body temperature >38°C or <35.5°C, leukocyte count >12,000 cells/mm3 or <4000 cells/mm3, purulent bronchial secretions, or reduced oxygenation [8]. **VAP** was defined as a pneumonia that was not in its incubation period before the initiation of mechanical ventilation (MV) and that developed >48 h after intubation. A **microbiological diagnosis** was ascertained by quantitative culture (≥10^3^ cfu/ml) of a respiratory sample obtained by protected telescopic catheter (PTC) [8–10]. Quality of the sample was assessed by the presence of leukocyte. The operating characteristics of the PTC were reported similar to those obtained from lung cultures. Several studies have shown that, once bacterial infection of the lung is clinically apparent, there are at least 10^4^ microorganisms/g of tissue. Several investigators have confirmed that, in pneumonia, pathogens are present in lower respiratory tract inflammatory secretions at concentrations of at least 10^5^ cfu/mL, and contaminants are generally present at less than 10^4^ cfu/mL [3]. The diagnosis threshold proposed for PTC and recommended by expert, is based on this concept. Because PTC collected between 0.001 and 0.01 mL of secretion, the presence of more than 10^3^ bacteria in the originally diluted sample (1mL) actually represented 10^5^ to 10^6^ cfu/mL for pulmonary secretions [3,11]. As recommended by all experts, the diagnostic threshold used to discriminate infection from colonization was 10^3^ cfu/ml or more when using quantitative culture of PTC[3,11].

VAP was considered **secondary to *S*.*aureus*** if isolates showed over 10^3^ cfu/ml, whether culture was mono or polymicrobial. Moreover, the accountability of *S*.*aureus* in VAP could also be retained by the presence of *S*.*aureus* as the sole pathogen identified in samples obtained from pleural drainage or LA echo-guided puncture.

**Abscess associated-VAP** was defined as the development of a LA or a necrotizing pneumonia during VAP. LA was defined on a CT-scan as the necrosis of the pulmonary tissue and formation of cavities containing necrotic debris or fluid, with or without gas, that was absent on the initial CT scan. It was distinguished from a pneumatocele identified as a round-shaped radiolucent lesion with a thick wall and ill-defined irregular margins. In case of an abscess, the vessels and bronchi are not displaced by the lesion, as they are by an empyema. Abscesses forms acute angles with the pleural surface. The formation of multiple, small (< 2 cm) abscesses is occasionally referred to as a necrotizing pneumonia. Since both LA and necrotizing pneumonia are the manifestations of a similar pathological process, and we used the same terminology for both.

All lung contusions were initially considered as a whole and then classified according to their severity. They were defined as (i) ground-glass opacification: a hazy increase in lung attenuation, with preservation of bronchial and vascular margins, (ii) consolidation, referred to as dense parenchymal opacification; whether patchy or diffuse, it usually refers to pulmonary parenchyma, which is entirely or almost completely airless.

**Clinical cure** was defined as the normalization of body temperature and the reduction of both pulmonary infiltrates and physical signs of pneumonia within the 7 days of antibiotics administration.

### Standard of care in the intensive care unit

#### Microbiological sampling

When pneumonia was suspected, a protected distal sampling was performed. In our unit, only sampling for clinical infection was used. Fiberoptical bronchoscopic examination was performed to each patient using a protocol previously described in detail [11]. The bronchoscope was introduced through a special adaptor and progressed through the bronchial orifice of a lung segment identified radiologically as the one containing the new infiltrate. The PTC (*combicath*^®^; *Plastimed*, *Prodimed*, *France*) was then moved to a subsegmental, peripheral position, after dislodging the distal plug to obtain lower airway secretions for microbial cultures. The tip of the protected inner catheter was vortexed vigorously in 1 ml of sterile 0.9% saline to ensure the full release of all material from the catheter.

#### Chest X-ray and CT-scan

All trauma patients had a body-scan at admission. During critical care stay, chest X-ray were realized routinely, about every 2 or 3 days depending on clinical evolution. An additional thoracic CT-scan was accomplished only in cases of complicated thoracic outcome and/or if a clinical or radiological suspicion of LA existed (e.g.: pneumonia with suspected abscess, pleural empyema, persistent pleural effusion despite thoracic drainage, rounded aerial image on chest X-ray). For each patient, the risk (transportation of a dependent patient) /benefice (therapeutic action) ratio of moving the patient to the radiology unit was weighted.

#### Mechanical ventilation

All mechanically ventilated patients followed a protocol to prevent VAP (head of bed elevation, control of tracheal tube cuff pressure, positive end-expiratory pressure, no systematic peptic ulcer disease prophylaxis, deep vein thromboprophylaxis, antiseptic oral care without oropharyngeal decontamination).

All patients in our unit were protectively ventilated to avoid as much as possible any lung injury. Tidal volume was set between 6 and 8 ml/kg of predicted body weight and targeting plateau pressure <30 cm H_2_O. Positive End Expiratory pressure was ≥ 5 cmH_2_O, depending on the severity of hypoxemia. The FiO_2_ was minimized when > 50%, targeting SaO_2_ between 88%-92% [12]. We modified ventilator settings primarily to achieve patient-ventilator synchrony. If this failed, we used the least amount of sedation required to achieve comfort and avoid unnecessary neuromuscular blockade.

#### Bacteriological specimens and investigations

Specimens were sent to the laboratory for direct examination after Gram staining, and culture. Specimens were immediately Gram-stained and graded for the presence or absence of polymorphonuclear leukocytes (PMN) and bacteria. Two serials 100-fold dilutions were made, and 0.1ml aliquots of the original suspension and each dilution were seeded on blood agar for aerobic and anaerobic cultures. Culture media included 5% sheep blood agar, chocolate agar (in 5% CO2), TSA, MacConkey agar and Schaedler broth. Inoculated media were incubated at 35–37°C, for 5 days. Standard methods were used for bacterial identification and susceptibility testing was performed by the agar dilution replicate plating method. Bacterial growth was expressed as colony-forming units per milliliter (cfu/ml) of the original saline suspension and considered numerically significant if ≥ 10^3^ cfu/ml.

Because of the retrospective design of this study, no genotypic identification of specific virulence factors was possible. Only routinely tested virulence factors were available (such as inoculum…). This search for PVL remained at the discretion of the physician.

Detection of PVL and mecA genes was achieved by PCR using the GenoType^®^ MRSA test system (*Hain Lifescience GmbH*, *Nehren*, *Germany*).

### Data collection

Patients were identified from the microbiology laboratory database combined with that of the ICU. Variables considered for each patient included in the study were the following: demographics (ie, age and sex); underlying comorbidities (ie, chronic heart disease, COPD, diabetes mellitus, HIV infection, corticosteroid use, alcoholism), clinical feature and severity of VAP (ie, symptoms related to sepsis, the development of septic shock, acute kidney injury, length of MV, ventilator settings, acute respiratory distress syndrome, lung compliance), treatment, results of laboratory analysis (complete blood count, biochemical analysis, blood culture and respiratory sample culture), and in-hospital outcome (ie, clinical or microbiological cure, MV weaning, mortality).

VAP-associated pathogens were recorded only if their inoculum was above the traditional PTC-threshold (≥ 10^3^ cfu/ml).

### Outcome parameters

Main discriminant parameter was the occurrence of LA during *S*.*aureus*-VAP. Secondary outcome variables were the details of the patients, the characteristics of VAP, the host risk factors for abscess formation, and finally *S*.*aureus* microbial particularity allowing to predict among ICU patients those who would develop or not LA.

### Statistical analysis

Statistical analysis was performed using IBM SPSS Statistics, Version 20.0 (Armonk, NY: IBM Corp.). Quantitative data were expressed as median [25th-75th percentile] and qualitative data as number (percentage). The Mann-Whitney U test was used to compare quantitative data. The Chi-squared test or the Fisher’s exact test was used, as appropriate, for qualitative data comparison. P value < 0.05 was considered significant.

## Results

**Fig 1:**
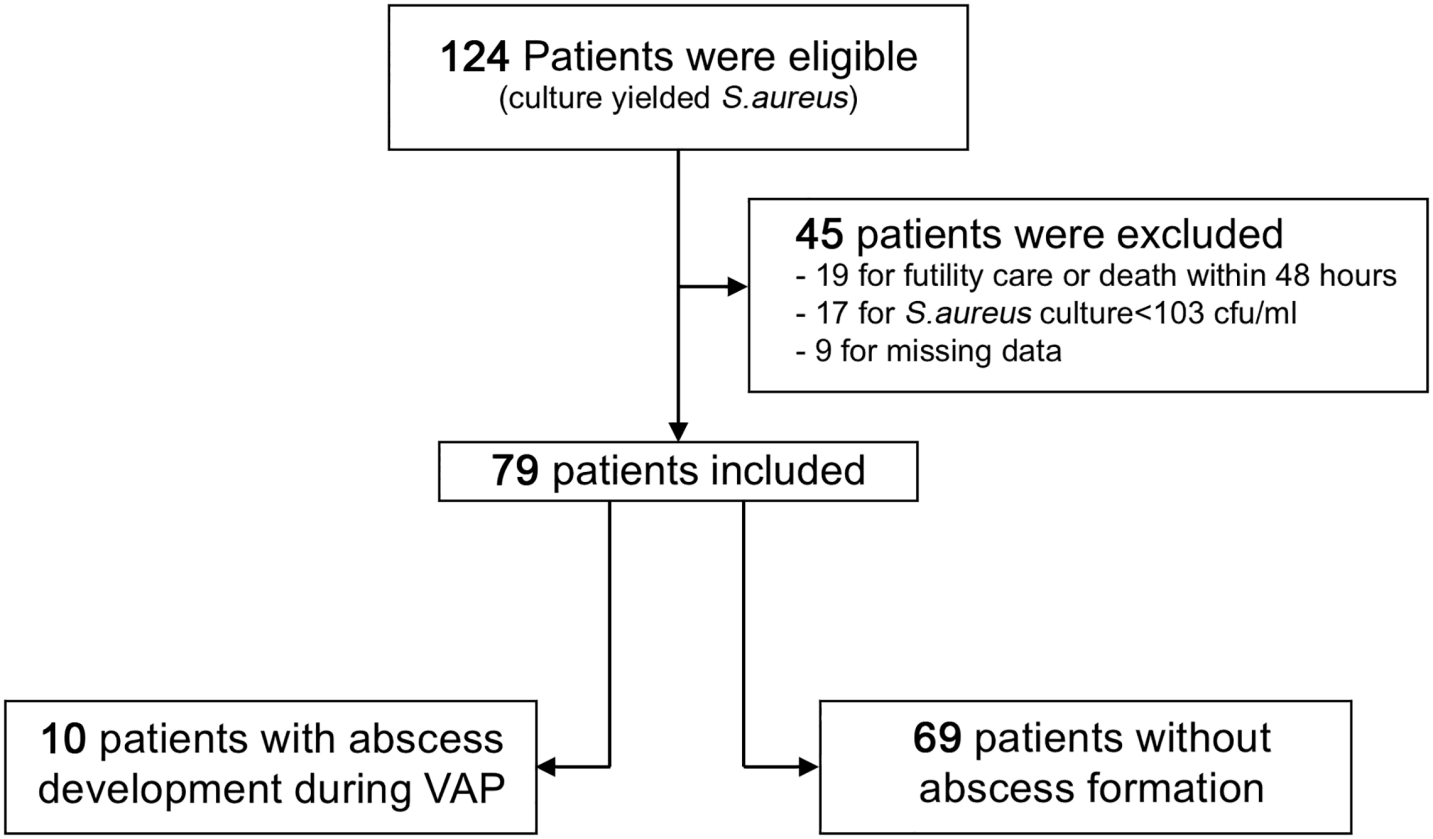
Flow chart of the study. Exclusion criteria: (i): pneumonias that were not associated with mechanical ventilation; (ii) patients with preexisting (ICU admission) major lung insult (e.g.; important emphysema or antecedent of lung abscess), (iii) patients suffering from α1-antitrypsin deficiency or cystic fibrosis, (v) VAP without microbiological documentation, (vi) all situations when futility of care within the first 48h following diagnosis was anticipated, (vii) and death within the first 48h. Cfu, colony forming unit; VAP, ventilator associated pneumonia.

Between January 2009 and July 2013, 124 patients were eligible. All presented VAP with *S*.*aureus* identified in pulmonary sampling (Fig 1). After exclusion criteria were applied, 79 VAP-patients showed *S*.*aureus* inoculum > 10^3^ cfu/ml threshold. Among these *S*.*aureus* VAP- patients, 10 developed LA (Fig 1). Data collected are reported in S1 File.

### Baseline characteristics

The details of the 79 *S*.*aureus* VAP-patients, as well as the characteristics of those who suffered LA are listed in Table 1. All patients were mechanically ventilated within the first 24h following admission. The patients were predominantly young male (35yr. [21–64], male 85%), presenting with severe trauma (trauma admission, 62%; SAPSII, 42 [32–52]). Underlying diseases and surgery were not different between patients suffering from LA (Abcess, n = 10) or not (NoAbcess, n = 69). However, abcess patients were significantly younger (p = 0.004) and had lower GCS upon arrival (p = 0.009) than NoAbcess. Median length of MV was 18d [8–23].

**Table 1:** Overall details of the patients and differential characteristics of those that suffered LA.

	Overall Details (n = 79)	*S*.*aureus*-VAP Patients		OR [95%CI]	p values
		Abcess (n = 10)	NoAbcess (n = 69)		
Male sex	67 (85)	10 (100)	57 (83)	-	0.35
Age (y)	35 [21–64]	20 [17–32]	45 [23–67]	-	**0.004**
Reasons for admission					
Trauma	49 (62)	9 (90)	40 (58)	-	0.42
Digestive surgery	5 (6)	0	5 (7)		
Vascular surgery	11 (14)	0	11 (16)		
Neurosurgery	7 (9)	1 (1)	6 (9)		
Medical	7 (9)	0	7 (10)		
Underlying diseases					
COPD	3 (4)	0	3 (4)	-	1
Tobacco	17 (22)	1 (10)	10 (23)	0.37 [0.04–3.13]	0.68
Immunodeficiency	2 53°	0	2 (3)	-	1
Diabetes mellitus	9 (11)	0	9 (13)	-	0.59
Chronic heart failure	11 (14)	0	11 (16)	-	0.34
Chronic renal failure	7 (9)	0	7 (10)	-	0.59
Gravity score at admission					
SAPSII	42 [32–52]	45 [34–53]	42 [32–52]	-	0.53
GCS	8 [5–15]	5 [3–7]	9 [6–15]	-	**0.009**
Length of ICU stay (d)	21 [9–29]	30 [19–45]	20 [8–28]	-	**0.038**
Duration of ATB treatment	7 [5–10]	10 [7–12]	7 [5–8]		**0.029**
Clinical cure	47 (59)	7 (70)	40 (58)	1.69 [0.4–7.1]	0.73
Death	30 (38)	3 (30)	27 (39)	0.67 [0.16–2.8]	0.73

NOTE. Data are no. (%) or mean [Q1-Q3]. Statistical analysis was done comparing the two groups (Abscess vs no abscess). ASA, American society of anesthesia; ATB, antibiotics; COPD, chronic obstructive pulmonary disease; GCS, Glasgow coma scale; ICU, intensive care unit; LA, lung abscess; OR, odds ratio; SAPSII, simplified acute physiologic score

### Ventilator-associated pneumonia characteristics

The 2 groups were similar. *S*.*aureus* related-VAP occurred shortly after ICU admission (medial 3 days from mechanical ventilation start), and represented the first episode of VAP during mechanical ventilation. They were not preceded by acute respiratory distress syndrome. *S*.*aureus* related-VAP were mostly monomicrobial (50%), with a significant inoculum (≥10^5^ cfu/mL).

### Host associated-factors with abscess formation during *S*.*aureus*-VAP

Groups were different regarding host associated-factors for abscess formation (Table 2). A previous episode of VAP did not increase the risk of LA, unlike the length of MV. Among trauma patients, the road traffic accidents significantly favored LA formation (p = 0.037). Head injury and chest trauma, were significantly associated to LA formation (p = 0.002 and p = 0.014, respectively). When considering chest trauma patients, identification of a pneumothorax at admission (requiring drainage) and pulmonary contusion were significantly associated with LA occurrence (p = 0.013 and p = 0.004, respectively). The need to place a chest tube at admission (during the 24 first hours) was significantly associated to LA formation (p = 0.004), but not later (0 vs 5 (7%), for LA and no LA group respectively). Pneumatocele was not associated with the risk of LA formation.

**Table 2:** Host risk factors for abscess formation.

	*S*.*aureus*-VAP Patients		OR (95% CI)	p values
	Abcess (n = 10)	NoAbcess (n = 69)		
Length of MV until VAP	3 [3–7]	3 [2–7]	-	0,74
≥ 1 VAP before	1 (10)	4 (6)	1.81 [0.81–18]	0.5
ARDS before	1 (10)	1 (1)	7.56 [0.43–131.62]	0.24
Patients on ATB at diagnosis	1 (10)	4 (6)	1.81 [0.18–18]	0.5
Road traffic accident	7 (70)	23 (33)	4.67 [1.1–19.74]	**0.037**
Head injury	10 (100)	34 (49)	-	**0.002**
Chest trauma	8 (80)	25 (36)	7.04 [1.39–35.77]	**0.014**
hemothorax	1 (10)	2 (3)	3.72 [0.31–45.31]	**0.034**
Pneumothorax	4 (40)	5 (7)	8.53 [1.8–40.55]	**0.013**
lung contusion	7 (70)	15 (22)	8.4 [1.93–36.48]	**0.004**
Ground Glass	0 (0)	6 (9)	-	1
Consolidation	7 70)	9 (13)	15.56 [3.39–71.35]	**0.0003**
Pneumatocele	2 (20)	6 (9)	2.62 [0.45–15.28]	0.27
Ribs fracture	5 (50)	16 (23)	3.31 [0.85–12.9]	0.12
Chest tube at admission	5 (50)	6(9)	10.5 [2.35–46.87]	**0.004**
Length of MV (d)	21 [18–34]	15 [8–21]	-	**0.04**

Note. Data are no. (%) or mean [Q1-Q3]. For lung contusion, only the more severe injury was reported. ≥ 1 VAP before, at least one ventilator-associated pneumonia before this *S*.*aureus*-VAP, but during the same hospital stay. ARDS before, means the same thing but for Acute Respiratory Distress Syndrome. Patients on ATB at diagnosis means ATB active on *S*.*aureus*. Chest tube means tube before abscess formation. Hemothorax and pneumothorax mean injuries presented at admission. ATB, antibiotics; MV, mechanical ventilation; VAP, ventilator-associated pneumonia.

Pattern of MV was similar between groups. Notably, comparisons day by day of tidal volume/kg of predicted body weight and plateau pressure were not significantly different (data not shown). No patient suffered endocarditis.

### *S*.*aureus* induced LA: Microbiological particularities

Not a single tested variable was significantly associated to abscess development (Table 3).

**Table 3:** Details of *S*.*aureus*-VAP risk factors for LA formation.

	*S*.*aureus*-VAP patients		p values
	Abcess (n = 10)	NoAbcess (n = 69)	
Antibiotics susceptibility			
Meticillin	10 (100)	63 (91)	1
Ofloxacin	10 (100)	62 (90)	0.59
Vancomycin	10 (100)	69 (100)	1
linezolid	10 (100)	69 (100)	1
Rifampicin	10 (100)	69 (100)	1
Cotrimoxazole	10 (100)	69 (100)	1
Inoculum (cfu/ml)			1
10^3^	3 (30)	11 (16)	0.88
10^4^	0 (0)	14 (20)	
10^5^	3 (30)	16 (23)	
10^6^	4 (40)	28 (41)	
Associated *S*.*aureus* bacteraemia	0 (0)	5 (7)	1
Absence of Gene for PVL*	7/7 (100)	2/2 (100)	1
VAP-associated pathogen			
none	6 (60)	34 (49)	0.36
*Haemophilus influenzae*	4 (40)	6 (9)	
*Streptococcus pneumoniae*	0 (0)	5 (7)	
*Streptococcus spp*.	0 (0)	1 (1)	
*Klebsiella pneumoniae*	0 (0)	6 (9)	
*Klebsiella oxytoca*	0 (0)	5 (7)	
*E*.*coli*	0 (0)	5 (7)	
*Others enterobacteriae*	0 (0)	6 (9)	
*Pseudomonas aeruginosa*	0 (0)	1 (1)	

Note. Data are no. (%) or mean [Q1-Q3]. *S*. *aureus* isolates were characterized by PCR for the presence of the PVL locus (lukS-PV and lukF-PV).

*For PVL variable, % is relative to the number of strain that were tested.

Cfu, colony forming unit; LA, lung abscess; PVL, Panton-Valentine Leucocidine; VAP, ventilator-associated pneumonia.

Resistance pattern of the strains were similar, and fully susceptible to major antibiotics.

Few variables were tested regarding any virulence pattern: inoculum, associated bloodstream infections (for *S*.*aureus* only) and carriage of PVL genes. When tested, PVL gene was never found, whatever the group. None were significantly different between groups.

Finally, VAP-associated pathogens known for their ability to form abscesses were tested; none were significantly different.

### Abscesses characteristics

**Fig 2:**
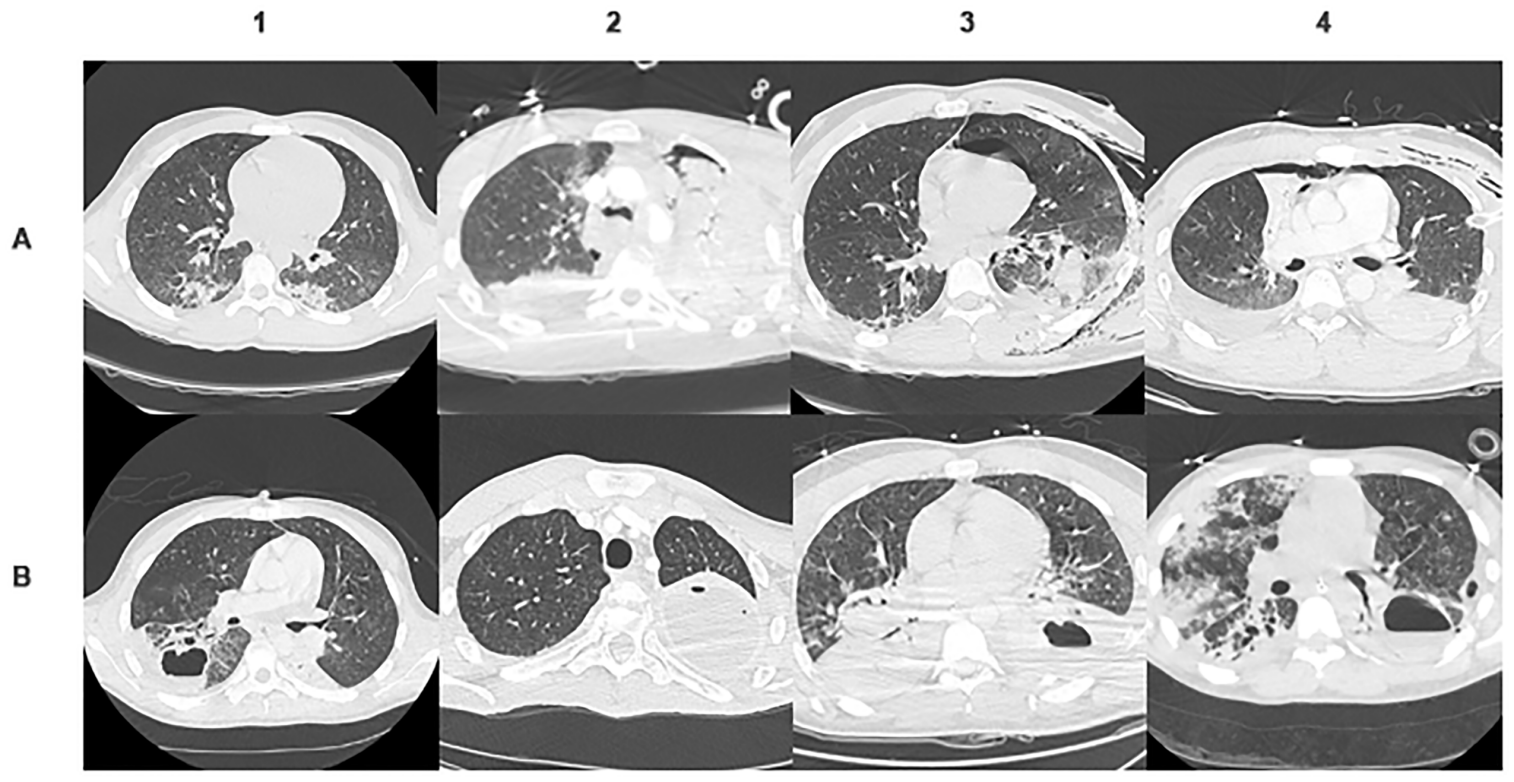
CT scan on admission (A) and under mechanical ventilation (B) of 4 patients presenting an abscess (1, 2, 3, and 4). We selected the most obvious lung abscesses. In the first place, CT scan illustrated that abscess occurred in the most contused area. Secondly, these images allowed us to appreciate the size and shape of the abscess. The thin-walled cavity and its surrounding consolidation appear.

The median length of time from admission to LA diagnosis was 13 days [8.7–23.2]. Focusing on the sub-population which developed LA (n = 10 patients), we observed that abscesses were multiple in 7 cases (≥2), bilateral in 5 cases, and measured more than 5 cm of major axis in 7 cases. Mostly, abscesses were located in the dependent areas of the lungs (9/10). On computed tomography all abscesses showed a thin-walled cavity surrounded by consolidation (Fig 2).

Focusing on spatial matching between traumatic lung injury and abscess location, we observed that the latter were localized in the most severely injured lung regions (Table 4). We do not report any ground-glass opacification, even if present when more severe injury coexists. But in these cases, abscesses were located in the consolidation areas.

**Table 4:** Concordance between initial spatial lung injury and abscesses location.

	Lung Contusion		Pneumatocele
	Ground-glass	Consolidation	
**Presence in abscess group (n = 10)**	0 (0%)	7 (70%)	2 (20%)
**Spatial matching with abscess**	-	100%	100%

Note. For lung contusion, it is the same patients who presented ground-glass and consolidation.

### Clinical course

LA were placed a chest tube at hospital admission in 50% of cases, as compared to 9% (p = 0.04) among those not developing LA. LA patients had a longer duration of mechanical ventilation (Table 1). Their treatment (using a combination of antibiotics including rifampicin) and hospital stay were longer (p = 0.029 and p = 0.038, respectively) (Table 1). Oesophageal endoscopy was performed in LA. Neither CT scan nor endoscopy evidenced oesophageal injury. LA did not affect the mortality rate.

## Discussion

We report a 13% LA incidence among *S*.*aureus* VAP in our trauma ICU patients. This particular feature combining a lung necrotic process associated with *S*.*aureus* pneumonia occurred in young men, mechanically ventilated after road traffic accident responsible for combined head and chest trauma resulting in severe lung injury requiring initial pleural drainage placement. We identified, among the patients that suffered LA concomitantly to *S*.*aureus* VAP, a strong link, both temporal and spatial, between pulmonary contusion (evolutive status and location). Interestingly, abscess formation was not associated to microbiological factors, especially *S*.*aureus* carriage of the PVL gene.

*S*.*aureus* is a pathogen that digs abscesses in the lung of patients. It is the most common etiologic germ responsible for LA in children [13], but its implication goes on as age grows. Indeed, the characteristic feature of *S*.*aureus*-NCAP led us to look for a particular pathogenicity of *S*.*aureus* strains that infected the lungs of our patients [4,14]. *S*.*aureus*-NCAP was described primarily in young people without any comorbidity, and was linked to the production of the Panton-Valentine leukocidin. CT scan showed a necrotizing pattern, with multiple confluent abscesses, almost bilateral, with interstitial infiltrate and pleural effusion [4]. Pediatric forms were more often associated to pneumothorax and pneumatocele [7]. Many features of *S*.*aureus*-NCAP were similar to those observed in case of *S*.*aureus*-VAP. Our patients developing abscesses were also young, without any comorbidity. Radiologic findings were quite similar, with an extensive necrosis and serious infiltrate. However, dynamic evolution differed radically resulting in relatively lower mortality rate in case of *S*.*aureus*-VAP as compared to that of *S*.*aureus*-NCAP, possibly linked to the fact that *S*.*aureus* did not carry PVL gene or exhibited other particular virulence factors we did not searched for [15]. Unfortunately, we have not realized genotypic identification of particular virulence factors such as the clonal complex, alpha-type phenol-soluble modulins, arginine catabolic mobile element, and mostly alpha-hemolysin [2,7,15]. Because of the retrospective design of this study, only routinely tested virulence factors were available. In terms of bacteriological data available (susceptibility pattern, inoculum, associated-pathogen, bacteriemias), we found no difference among the patients that suffered or not LA. Susceptibility patterns were similar between groups, and interestingly our strains were fully susceptible: all the tested strains were not carrying the PVL gene [9]. Based upon litterature, presence of PVL toxin can be suspected in the case of a particularly aggressive pneumonia occurring in a specific type of patients. Thus, the search for PVL gene was more frequent in the abscess group (still remaining at the discretion of the physician).

As reported by some authors, the presence of *S*.*aureus* in the respiratory tract can lead to a wide range of outcomes, from asymptomatic colonization to fulminant invasive disease, and the host immune response is a significant determining factor of the outcome [2,16]. Analysis of LA associated-factors related to *S*.*aureus* was disappointing in our series of patients. We overlooked at risk factors related to aspiration pneumonia that shared similar pathophysiology. The *S*.*aureus*-VAP we evidenced occurred early after admission (d+3), and patients with abscess formation were significantly more comatose (5 [3–7] vs 9 [6–15], p = 0.009). Aspiration pneumonia resulting in LA has been demonstrated to develop in altered mental status patients, in case of large and polymicrobial inoculum [17]. According to recent studies, incidence of anaerobes decreased over time [17]; cultures of our respiratory samples were indeed free from anaerobes. Regarding aerobes associated-pathogens, we focused on the most common, which are known to lead to abscess formation [17,18]: *K*.*pneumoniae*, *E*.*coli*, *H*.*influenza*, *P*.*aeruginosa* and *Streptococcus* pyogenes (including *S*.*pneumoniae*). Our statistical analysis failed to describe any association, whichever responsible microbe, despite an incidence of pathogen-association similar to those reported in the literature [17]. Anyway, our *S*.*aureus*-VAPs were less polymicrobial than expected. On the opposite, in more than 90% of the cases of LA described in the literature, polymicrobial bacteria were found [19]. In our cohort, inoculi were similar between groups and did not appear to have a role in abscess formation. This is consistent with more modern theories that highlighted the fact that pneumonia results from a complex interaction between host and inoculum, as opposed to inoculum alone [20]. A large proportion of healthy subjects, with a normal mental status, aspirates during sleep [21] without any general consequence or respiratory morbidity [20]. Therefore, inoculum size alone is not sufficient to explain lung insult intensity. Authors studying LA reported the same findings: the immune system has a significant role in abscess pathogenesis [18].

Our patients with abscess were young, free from any comorbidity, immunocompetent and non-smoker in most case (n = 8). Hypoxemic situation and VAP incidence before *S*.*aureus*-VAP occured were similar in both groups. *S*.*aureus*-VAP patients developing LA shared similar causes of ICU admission and SAPS II score, when compared to those without abscess. Therefore, another factor may have contributed to our findings. We believe that the major predisposing factor that has contributed to LA formation is certainly related to the severity of the initial lung injury. Indeed, our results highlight the strong association between the severity of the chest trauma and the formation of LA. Similarly to brain trauma intensity, the patients developing LA showed a significant worse initial GCS score and most of them had high velocity road traffic accidents. The role of kinetic energy transfer during trauma is a well-known risk factor for insult severity. As already reported, the combination of head and chest trauma was more frequently associated to the highest kinetics and energy transfer situations[22]. Our data confirms such observations showing that *S*.*aureus*-VAP patients developing LA suffered from more lung parenchymal insults than those who did not. Pneumothorax and lung contusions were, respectively, significantly more frequent and severe, before abscesses developed in cases of *S*.*aureus*-VAP. Contusion is the most common type of lung injury in blunt chest trauma, but is not known to be associated to abscess formation [23,24]. Focusing on the severity of contusion, we report that the most severe contusions (consolidation) were strongly associated to abscess development in case of *S*.*aureus*-VAP. Moreover, our results indicate a perfect spatial matching between initial contusion and final abscess localization. All abscesses erupted in the initial consolidation areas. We can hypothesize that the first parenchymal insult was located in the lung areas where maximum energy had been transferred and dissipated during trauma leading to contused areas. Secondary insult could results from mechanical ventilation’s consequences on a low compliance, fragile, unstructured lung area suffering from significant surfactant activity deficiency [25,26]. The third insult could be initiated by bacteria (ie, *S*.*aureus)* reaching the lung contused sites (via different possible pathways such as: direct lung aspiration before ventilation, the subglottic space, along the tracheal tube, directly from the lung microbiota, the blood, or others). The combination of these successive attacks on the lung parenchyma, associated with abnormal baro-trauma induced-immune system response, has certainly promoted abscess formation in the most weakened lung areas.

Obviously, our study presented some limitations. First, we excluded LA not caused by *S*.*aureus*. Future research is required to evaluate all LA occurring after chest trauma. Second, we could be argued that it is complex to clearly distinguish abscess from pneumatocele, as reported in ventilator-associated injury [27]. However, our lung insults presented a thick inflammatory wall with ill-defined irregular margins and were mostly located in the deepest areas of the lung. Third, our study was retrospective, and therefore presented with some inherent bias. Indeed, length of both MV duration and ICU stay were increased in the group of *S*.*aureus*-VAP patients that developed LA when compared to those that did not. The reason for this difference seems obvious. Indeed impairment of both respiratory mechanics and oxygenation parameters associated with LA formation and development have certainly lengthened the need for mechanical ventilation, and tracheal tube maintenance (clearing of secretions) resulting in longer ICU stay. The fact that in both groups, patients declared *S*.*aureus*-VAP at a similar moment, favors this hypothesis.

## Conclusions

We observed that LA associated to *S*.*aureus* VAP were mostly, multiple, bilateral and situated in the dependent areas of the lung. LA associated to *S*.*aureus* VAP concerned predominantly young males ICU patients, mechanically ventilated after high kinetic road traffic accident, responsible for combined severe head and chest trauma. The most serious contused areas matched perfectly with abscess location in case of *S*.*aureus* VAP. The causes of abscess formation secondary to *S*.*aureus* VAP are multiple, but not linked to specific virulence factors of *S*.*aureus*.

## Supporting information

S1 File Data file. (CSV)
